# Comparison of praziquantel pharmacokinetics and tissue distribution in fresh and brackish water cultured grass carp (*ctenopharyngodon idellus*) after oral administration of single bolus

**DOI:** 10.1186/s12917-015-0400-2

**Published:** 2015-04-01

**Authors:** Xinyan Xie, Yini Zhao, Xianle Yang, Kun Hu

**Affiliations:** College of Aquaculture and Life Science, Shanghai Ocean University, No. 999, Huchenghuan Road, Shanghai, 200090 P.R. China

**Keywords:** Praziquantel, Pharmacokinetics, Grass carp, Salinity, Tissue distribution, Elimination

## Abstract

**Background:**

Praziquantel (PZQ) is an effective pesticide against monogeneans. Its pharmacokinetics in fish may be affected by water environment and temperature. The present study was designed to compare the pharmacokinetics, tissue distribution, and elimination of PZQ in freshwater-acclimated grass carp and brackish water cultured grass carp. Plasma and tissue PZQ concentrations were determined after a single 10 mg/kg oral PZQ dose.

**Results:**

The datas of plasma and tissues drug concentration was calculated by the software SPSS 13.0. According to the One-Way ANOVA, the results showed that the salinity had a significant effect on the drug concentration of plasma (*p* < 0.01), muscle (*p* < 0.01), liver (*p* < 0.01) and kidney (*p* < 0.01) in the all sampling time points between the brackish water grass carps and the freshwater grass carps, wherein, PZQ plasma and tissue concentrations in the brackish water group were constantly lower than that in the freshwater group. The peak PZQ levels of plasma, muscle, liver, and kidneys in the brackish water group were 0.76 μg/ml, 0.51 μg/g, 2.7 μg/g, and 2.99 μg/g, respectively; and that in the freshwater group were 0.91 μg/ml, 0.62 μg/g, 3.87 μg/g, and 3.39 μg/g, respectively. The elimination half-lives (t_1/2β_) in plasma and all tissues of the freshwater group were significantly longer than that in the brackish water group. The elimination half-lives (t_1/2β_) of plasma, muscle, liver and kidneys in brackish water grass carps were 56.46, 36.17, 15.31, and 132.64 h, respectively; and that in the freshwater grass carps were 71.15, 44.88, 23.86, and 150.23 h, respectively.

**Conclusion:**

These findings indicate that water environment affects the tissue distribution and elimination of PZQ in grass carps, the elimination in brackish water grass carps is more rapid than that in fresh water grass carps and tissue concentrations of brackish water grass carps are lower than that in freshwater grass carps after orally administrating the same dosage at the same water temperature. We speculate that the main excretion pathway of the drug is through renal elimination, and the decreased kidney function in brackish water grass carps is likely responsible for the considerable difference in pharmacokinetics between the two groups of grass carps.

## Background

Praziquantel (PZQ) is widely used as a chemotherapeutic agent for treating helminths in captive fish. It is effective against monogeneans that infect the gills, skin, and branchial cavities [1-13], larval and encysted digeneans infecting the eyes [14] and skin [15], as well as intestinal cestodes [16] of teleosts and elasmobranchs.

Grass carp (*Ctenopharyngodon idellus*) is an economically important species of farmed fish. In China, grass carp has a widespread breeding distribution scope, covering different salinity areas in the eastern and northwestern regions, and freshwater areas of the central and southern regions. For example, the water salinity of Lian Yungang in Jiangsu province is 3.6‰ and that of Tachen Island in Zhejiang province is 5.8‰ [17]. Several studies have indicated that environmental water salinity can influence the accumulation and elimination of chemicals in fish [18-22]. The difference in pharmacokinetics influences the effectiveness of PZQ in aquaculture, especially for treating monogenean infection in brackish water cultured grass carps.

Chemical/drug pharmacokinetics in aquatic animals can be affected by various environmental factors, such as water temperature, PH, and salinity [23]. However, the effect of salinity is often overlooked, especially in freshwater fish. Moreover, in order to improve grass carp muscle quality, the fish are often raised in brackish water or placed into brackish water for a period of time before listing [24]. Considering that grass carp are cultured in both freshwater and brackish water, it is necessary to investigate possible tissue distribution and praziquantel elimination differences in grass carp under different water environments. We believe that this study would help better formulate effective PZQ regimens in treating monogenean infections of fish.

## Methods

### Chemicals and reagents

Praziquantel (2-cyclohexyl-carbonyl-4-oxo-1,2,3,6,7,11b-hexahydro-4Hpyrazino[2,1-a] isoquinoline) (99.9% purity) is used as an analytical standard and was purchased from Sigma-Aldrich (St. Louis, MO, USA); PZQ (purity 98%) used in the pharmacokinetic study was purchased from Feng Hua Pharmaceutical Co., Ltd., Hebei. HPLC-grade acetonitrile was purchased from Merck (KGaA, Germany); and all the other chemicals were of analytical grade.

### Animals

All the experiment fish precedures were reviewed and approved by the Institutional Animal Care and Use Committee at Shanghai Ocean University at Shanghai Ocean University. According to the Shanghai ocean university animal health guidelines for animal care and experimentation. About 120 six month age Grass carps (*Ctenopharyngodon idellus*, weighing 80 ± 5.6 g) were obtained from Nantong Fisheries Farm (Yancheng, China) and were randomly divided into two groups: freshwater group and brackish water group and number of fish in each group was 60. The two groups of fish were brought in the laboratory and placed in two ponds (1 m^3^). The brackish water group was gradually acclimated to brackish water with 3.5‰ salinity for five days; circulating water (the salinity for the freshwater and brackish water groups were 0 and 3.5‰, respectively) and oxygen were continuously supplied using an inflation pump. Before the experiment, the two groups were acclimated in rearing tanks for 10 days at 22 ± 1°C. During acclimation, they were fed twice daily with a drug-free commercial diet to apparent satiation, then starved for 24 h before drug administration.

### Drug administration

To prepare the PZQ dosing solution, PZQ was dissolved in 5 mL of ethanol and mixed with water to achieve a final concentration of 2 g/L. The fish were given 5 μL/g of the PZQ solution via gavage using a stomach tube -- with the final PZQ dosage being 10 mg/kg. After oral drug administration, each fish was placed in an observation tank for five minutes for possible drug regurgitation; regurgitated fish were excluded from the analysis.

### Sample collection

Blood sampleswere collected from the tail sinus of five fish at various time points (before dosing; and 0.25, 0.5, 1, 3, 6, 12, 24, 48, and 96 h after dosing); then, five fish in each sampling time point were killed by breaking their spine. Muscle, liver, and kidney samples were taken, immediately frozen, and stored at −20°C until analysis.

### Sample preparation

The analytical procedure for PZQ analysis was modified according to the report of Jing Yao [25]. In brief, the plasma (1 mL) or ground tissue samples (1 g) were separately placed into 50-ml centrifuge tubes, and mixed with 3 ml of ethylacetate using a Vortex vibration meter (Thermo Fisher Scientific, USA) for three minutes. After 10 minutes of centrifugation at 4,500 rpm, the supernatant was removed and transferred to a 15 ml centrifuge tube. The extraction step was repeated once. The combined ethyl acetate extracts were evaporated at 45°C to dryness using the rotary evaporation apparatus (Eppendorf, Germany). Residues were reconstituted with 1 ml of HPLC mobile phase, in which 2 ml of hexane was added. The mixture was vortexed for three minutes and centrifuged at 8,000 rpm for two minutes. The supernatant was removed and transferred to a 2-ml centrifuge tube; then 20 μL was injected onto the HPLC system, as described below.

### Chromatographic conditions

We used an Agilent (HP1100) HPLC system with a fluorescence detector. The separation was performed on a Zorbax XDB-C18 column (4.6 × 150 mm internal diameter, 5-μm particle size; Agilent Technologies, USA) using an isocratic mixture of acetonitrile: water (50:50, v/v) as the mobile phase at a constant flow rate of 1 ml/min. Injection volume was 20 μL, optical maser wavelength was 265 nm, emission wavelength was 280 nm, and column temperature was 25°C.

### Pharmacokinetic analysis

Pharmacokinetic analysis was performed using the DAS 3.0 program; and pharmacokinetic parameters were analyzed based on the classical compartmental analysis for plasma and tissue concentration-time data. The following pharmacokinetic parameters were estimated: C_max_ (peak concentration), t_1/2α_ (half-life of the absorption rate constant), t_1/2β_ (half-life of the elimination rate constant), AUC_(0-t)_ (area under the concentration-time curve from time zero to t), and CL/F (total body clearance).

### Statistical analysis

Statistical analysis between the two groups was completed using the SPSS 13.0 program.

## Results

### Analytical method validation

The analytical procedure for PZQ analysis was modified according to the report of Jing Yao [25]. A linear calibration curve over the 0.05 – 20 μg/ml PZQ range was established, yielding a correlation coefficient exceeding 0.9994. The drug concentration of the samples above the upper calibration limit was diluted with blank plasma and supernatants of the tissue samples. The results showed that all inter-day and intra-day coefficients of variation were below 3.34%; while tissue sample accuracy ranged from 74% to 85%, and mean recovery was above 76%. Based on a signal-to-noise ratio > 3, the lower limits of detection (LOD) and quantitation (LOQ) for this assay were 0.05 μg⁄mL.

### Pharmacokinetic analysis

**Figure 1:**
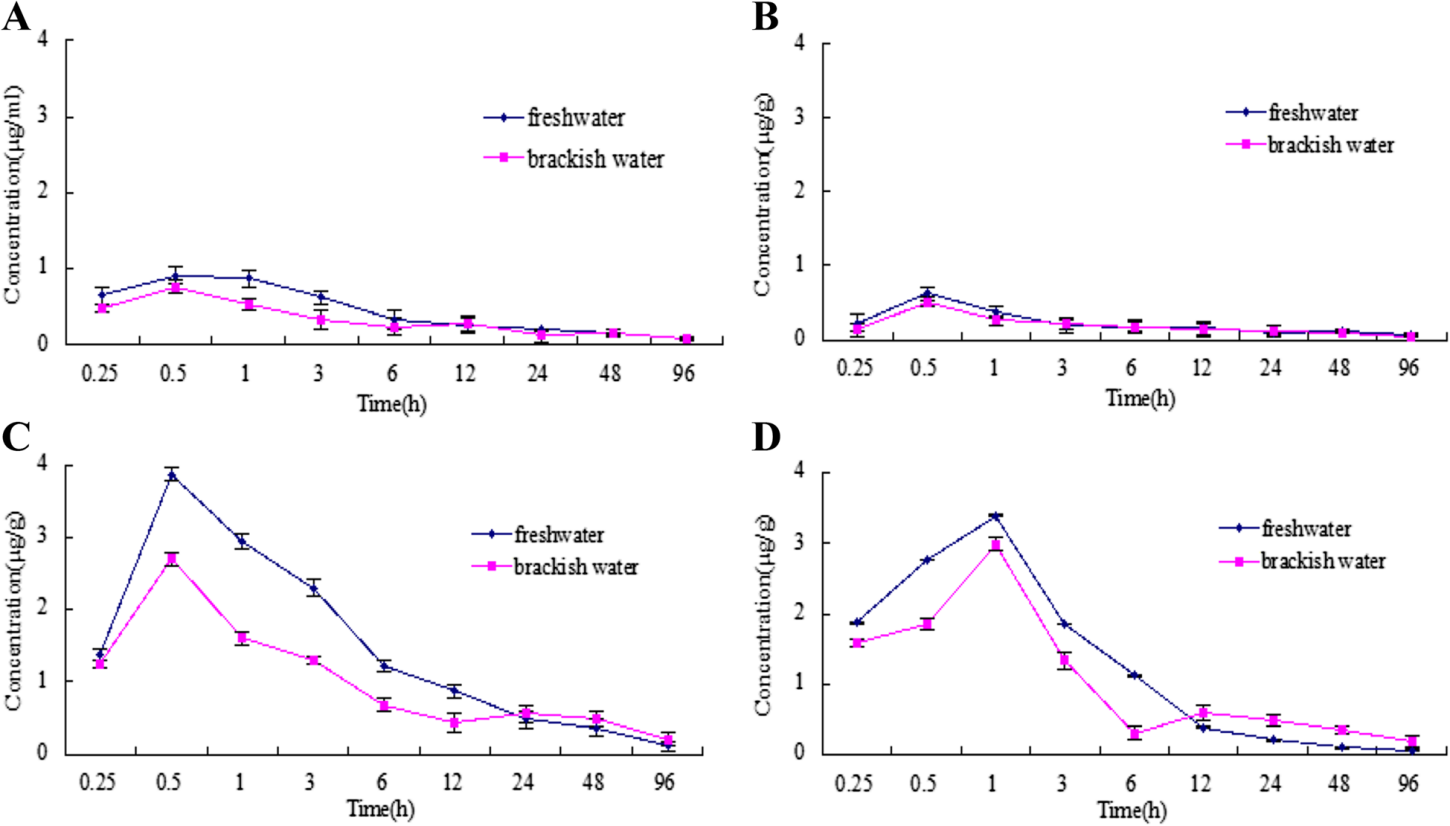
**Praziquantel in plasma and tissues of freshwater and brackish water grass carps after oral administration.** Standard deviation is given at vertical bars. (n = 5, The mean ± SD). (**A**: plasma; **B**: muscle; **C**: liver; **D**: kidney).

The PZQ concentration-time curves are shown in Figure 1. The pharmacokinetic parameters are shown in Table 1. The plasma, muscle and liver of freshwater group grass carp and the plasma, muscle and kidney of brackish water group PZQ concentration-time profiles were best described by a two-compartmental open pharmacokinetic model with first-order absorption. The kidney of freshwater group and liver of brackish water group PZQ concentration-time profiles were best described by a three-compartmental open pharmacokinetic model. The datas of plasma and tissues drug concentration was calculated by the software SPSS 13.0. According to the One-Way ANOVA, the results showed that the salinity had a significant effect on the drug concentration of plasma (*p* < 0.01), musle (*p* < 0.01), liver (*p* < 0.01) and kidney (*p* < 0.01) in the all sampling time points between the brackish water grass carps and the freshwater grass carps.

**Table 1:** **Pharmacokinetic parameters of Praziquantel in grass carp (n = 5)**

		**Plasma**		**Muscle**		**Liver**		**Kidney**	
**Parameter**	**Unit**	**Fresh water**	**Brackish water**	**Fresh water**	**Brackish water**	**Fresh water**	**Brackish water**	**Fresh water**	**Brackish water**
C_max_	ug/(g or ml)	0.91	0.76	0.62	0.51	3.87	2.70	3.39	2.99
T_max_	h	0.5	0.5	0.5	1	0.5	0.5	1	1
t_1/2α_	h	3.25	1.85	0.31	1.12	0.35	2.69	0.76	2.87
t_1/2β_	h	71.15	56.46	44.88	36.17	23.86	15.31	150.23	132.64
t_1/2Ka_	h	0.16	0.028	0.058	0.57	0.15	0.031	0.307	0.64
V1/F	L/kg	23.57	30.15	21.53	22.22	28.38	97.33	34.30	87.17
CL/F	L/h/kg	0.755	1.042	1.961	0.974	0.481	0.308	1.878	0.350
AUC_(0-t)_	μg/L⋅h	17.951	14.614	8.096	6.865	34.984	44.047	10.411	38.663
AUC_(0-∞)_	μg/L⋅h	26.464	19.189	10.196	20.515	41.547	64.823	10.645	57.132
K10	1/h	0.064	0.035	0.091	0.044	0.17	0.048	0.34	0.16
K12	1/h	0.235	0.212	1.574	0.474	1.469	0.113	2.034	0.754
K21	1/h	0.104	0.14	0.548	0.103	0.355	0.036	0.245	0.042
Ka	1/h	4.201	25.183	11.951	1.203	4.537	22.106	2.26	1.082

C_max_, the peak concentration in plasma; T_max_, the time point of the drug’s maximum plasma concentration; t_1/2α_, distribution half-life of the drug; t_1/2β_, elimination half-life of the drug; t_1/2Ka_, absorption half-life of the drug; V1/F, extensive apparent volume of the central compartment; CL/F, total body clearance of the drug; AUC_(0-t)_, area under the concentration–time curve from 0 h to t; AUC_(0-∞)_, area under the concentration–time curve from 0 h to ∞; K12, K21, first-order rate constants for drug distribution between the central and peripheral compartments; K10, elimination rate constant from the central compartment; Ka, absorption rate constant.

### PZQ distribution and elimination

Tissue concentrations are shown in Figure 1. The absorption of PZQ in freshwater grass carps was essentially the same as the brackish water carps. There were no significant differences in T_max_ of plasma, liver, and kidneys between the two groups.

The mean peak levels in plasma, muscle, liver and kidneys of the brackish water grass carps were 0.76, 0.51, 2.7, and 2.99 μg/g, respectively; which were lower than that in the freshwater grass carps (0.91, 0.62, 3.87, and 3.39 μg/g, respectively). In most sampling time points, the plasma and tissue PZQ levels in brackish water grass carps were lower than that in the freshwater grass carps.

The plasma and tissue PZQ elimination half-lives (t_1/2β_) in brackish water grass carps were shorter than that in the freshwater grass carps (Table 1). The PZQ elimination half-lives (t_1/2β_) in plasma, muscle, liver and kidneys of the brackish water grass carps were 56.46, 36.17, 15.31, and 132.64 h, respectively; and the figures for freshwater grass carps were 71.15, 44.88, 23.86, and 150.23 h, respectively. These findings indicated that PZQ elimination in brackish water grass carps was more rapid than that in freshwater grass carps.

## Discussion

Monogeneans can rapidly multiply in intensive farming facilities [26], necessitating early diagnosis, as well as rapid and effective treatment, to prevent benign infections from becoming pathogenic. PZQ is a useful chemotherapeutic against helminths of captive fish. There is a need for examining factors that affect its pharmacokinetics in order to minimize the potential development of anthelmintic resistance caused by extended exposure of parasites to subcurative doses [27].

Salinity conditions play an important role in pharmacokinetics and fish tissue residues. For example, oxolinic acid presents in lower concentrations for a longer period of time in seawater teleosts than freshwater teleosts [17]. Ishida [19] reported that oxolinic acid is excreted more slowly in freshwater trout than in seawater trout. Abedini *et al*. [20] suggested that freshwater trout may be used as a salmonid model to study oxytetracycline pharmacokinetics in seawater salmon; however, drug elimination and clearance rates in freshwater trout are remarkably lower than that in seawater salmon. Feng *et al*. [21] demonstrated that tissue drug concentrations of seawater tilapia are lower than that in freshwater tilapia; and the elimination of florfenicol in seawater tilapia is more rapid than that in freshwater tilapia. Even though grass carps could live in both fresh and brackish waters, possible PZQ pharmacokinetic differences between freshwater-acclimated grass carps and brackish water grass carps have never been reported.

In the present study, the datas of plasma and tissues drug concentration was showed that the salinity had a significant effect on the drug concentration of plasma (*p* < 0.01), muscle (*p* < 0.01), liver (*p* < 0.01) and kidney (*p* < 0.01) in the all sampling time points between the brackish water grass carps and the freshwater grass carps. The plasma PZQ concentration-time curve patterns were similar in the two groups, which could be best described by a two-compartmental open pharmacokinetic model with first-order absorption.

The CL/F is the total body clearance of the drug and the t_1/2β_ is the elimination half-life of the drug. The two pharmacokinetics parameters can describe how quickly the drug is eliminated from the fish. The CL/F of the freshwater group and brackish water grass carp plasma were 0.755 and 1.042 L/h/kg respectively. The elimination half-lives (t_1/2β_) of plasma in freshwater water grass carps and brackish grass carp were 71.15 and 56.46 h, respectively. Compare to the freshwater grass carps, the brackish water grass carps have more rapidly drug clearance and shorter elimination half-lives when the grass carp cultured in the low salinity water. The water salinity change will influence the regulation of osmotic pressure in the grass carps. Even though seawater salinity is greater than brackish water, the results indicated that osmoregulation still existed in brackish water fish, and that their drug excretion pathway may be altered. The results were consistent with previous similar studies [18,20,21].

K12 and K21 were the first-order rate constants for drug distribution between the central and peripheral compartments. The datas display that the K12 and K21 of the brackish water group grass carp were lower than that of freshwater group in most tissues such as liver and kidney. This phenomenon indicated that the salinity water could decrease the drug distribution rate between the central and peripheral compartments.

In the most sampling time points, the liver drug concentrations of the brackish water group are lower than that of freshwater group which water has no salinity; indicating that water salinity can affect drug metabolism in the liver, and that the drug may be eliminated through other drug excretion pathways, such as branchial excretion. Sohlberg *et al*. [28] reported that gill excretion occurs in seawater fish; Atlantic salmon excrete approximately 60% of administered flumequine through the gills in seawater. Feng *et al*. [21] reported that branchial excretion competence in seawater fish may be attributed to active drinking for osmoregulatory purposes of seawater tilapia; possibly resulting to the rapid excretion of the drug and its metabolites or their ionized complexes via the branchial chloride cells.

Kidneys are the main organs that excrete drugs and its metabolites. When the water has no salinity, the PZQ concentration in kidney was gently rising in the previous time points. Strangely, the PZQ concentration of brackish water grass carp kidneys slowly increased between 0.25 and 0.5 h after dosing, which peaked between 0.5 and 1 h; indicating that water salinity can also affect brackish water fish kidneys due to osmoregulation result in kidney weakening function. For grass carps in brackish water, the drug and its metabolites are likely to stay in the body and be reabsorbed by entero-hepatic cycling to some extent, retarding drug elimination due to the decrease in urine output through osmoregulation. When the renal drug concentration of brackish water grass carps slowly increased to its peak concentration between 0.25 and 0.5 h after dosing, the drug concentrations of the other tissues decreased. This phenomenon illustrated that the drug in the surrounding tissues entered the kidneys at that time, increasing the kidney drug concentration to its peak concentration; which again rapidly declined after micturition.

## Conclusion

In summary, our results indicate that salinity level greatly impacts the accumulation and tissue distribution of PZQ in grass carps, and that its elimination in brackish water grass carps is more rapid than that in fresh water grass carps. Further, tissue concentrations of brackish water grass carps are lower than that in freshwater grass carps, after orally administrating the same dosage at the same water temperature. We speculate that the main excretion pathway of the drug is through renal elimination, and the decreased kidney function in brackish water grass carps is likely responsible for the considerable difference in pharmacokinetics between the two groups of grass carps.
